# Preparation of Nanosilver and Nanogold Based on Dog Rose Aqueous Extract

**DOI:** 10.1155/2014/658935

**Published:** 2014-01-06

**Authors:** Jolanta Pulit, Marcin Banach

**Affiliations:** Cracow University of Technology, Warszawska Street, 24, 31-155 Cracow, Poland

## Abstract

This paper describes a process of obtaining nanosilver and nanogold based on chemical reduction using substances contained in the aqueous extract of dog rose (*Rosa canina*). The resulting products were subjected to spectrophotometric analysis (UV-Vis), and testing of the nanoparticles' size and suspension stability was carried out by measuring the electrokinetic potential, *ζ*, via dynamic light scattering (DLS). Solid samples were imaged by scanning electron microscopy (SEM). The obtained data were given to statistical analysis in order to illustrate the properties of the suspension depending on the values of the input parameters: metal salts concentration, pH of the reaction mixture, and process temperature. In the course of the work, samples of nanosilver and nanogold were obtained, which were stable for over two months and which had a monomodal particle size distribution.

## 1. Introduction

Nanosilver and nanogold are considered two of the most useful commercial products belonging to the group of nanomaterials. Recently their use has reached one of the highest levels of cost-effectiveness [1]. Many years of research have contributed to the confirmation of the centuries old argument that silver and gold slow down the functioning of bacteria. Therefore, in the daily exposure of animal organisms to microorganisms such as bacteria, viruses, or fungi, nanosilver and nanogold play a key role in antiseptic action [2]. Medicine, nursing, cosmetology, dentistry, and the construction industry belong to areas that take advantage of the benefits of nanosilver and nanogold to the greatest extent [3].

There are many known methods that are described in detail for the synthesis of nanosilver and nanogold. These include, among others: chemical reduction [4, 5]. This can be replaced with a procedure that is in accordance with the principles of “clean technologies.” With the development of nanotechnology, ecological methods have become an alternative to conventional processes that are not always compatible with the principles of green chemistry and are therefore environmentally nonindifferent [6]. The possibility of reducing the need to use substances that pose a potential threat to the environment is an important feature of ecological methods. The low cost of reduction is an additional property, as the process does not require the involvement of specialised laboratory equipment. The simplicity of the whole process is an additional feature [5, 7–10].

An important issue is the provision of a process for the synthesis of nanosilver or nanogold that is environmentally friendly. In order to better comply with the principles of “green chemistry,” compounds are sought that satisfy both roles, for stabilising and reducing substances. Limiting the amount of raw materials reflects the ideas of pro-ecological technologies. Methods for the preparation of metal nanoparticles using plant extracts are known in the literature. *Rhizophora mucronata *[11], *Chenopodium album *[12], *Jatropha curcas* [13], *Lawsonia inermis* [14], or black tea leaves [15] were used for this purpose.

The innovative concept of obtaining gold and silver nanoparticles by chemical reduction using nontoxic substances exhibiting both reducing and stabilising properties has been developed.

The current state of knowledge allows the prediction of the compounds which are derivatives or analogues of the original substance, and which are characterised by similar properties. The literature describes methods of carrying out the reaction for obtaining silver nanoparticles based on chemical reduction using a substance acting as both the reducer and stabiliser. Sodium citrate and gallic acid are such known substances [16]. By analysing the molecular formula of gallic acid (3,4,5-trihydroxybenzoic acid)—C_6_H_3_(OH)_3_COOH—it can be assumed that a compound consisting of similar atomic bonds will be characterised by analogous properties to those of gallic acid. Ellagic acid molecules are a dimer of gallic acid; both of these acids belong to a major group of compounds, polyphenols, which due to the presence in their structure of hydroxyl groups can be converted into a form that allows the transfer of charges and thus to act as a reducing agent [17–19].

It was decided to use aqueous extracts of selected parts of a plant in order to obtain a substance with both reducing and stabilising properties. In this work the aqueous extract of dried dog rose was used. This plant is a valuable source of different types of antioxidant. Above all it contains a significant amount of ascorbic acid [20, 21]. This plant is sometimes grown specifically for the purpose of obtaining ascorbic acid through other than synthetic means. The extract derived from dog rose is thus a natural source of antioxidant compounds, as confirmed by a number of studies conducted by different academic units [22–24]. Ascorbic acid, which is mainly present in the fruit of dog rose, is able to transform oxygen from the air into water. Of course the reaction is facilitated by the presence of metal ions and light radiation. The fact that ascorbic acid molecules can be oxidised to dehydroascorbic acid is relevant, and it is a valuable reducing compound and can reduce the oxidation degree of many chemical moieties [25, 26]. Apart from its valuable antioxidant properties, ascorbic acid, commonly known as vitamin C or food additive E300 in the food industry, acts as a preservative.

As other authors provide, one must be aware of the presence of ascorbic acid, polyphenolic compounds, and anthocyanins in the *Rosa canina* extract [23, 27–31]. Dog rose fruits are a natural source of polyphenols, including ellagic and gallic acids, and contain significant amounts of anthocyanins belonging to flavonoids. Ellagic acid is a compound known to have antioxidant properties [32]. Its antioxidant properties have been confirmed and documented in many scientific studies, including those relating to anti-tumour research and mute disease [33]. The fruits of dog rose also contain another compound from the group of polyphenols, gallic acid. Due to the fact that its structure includes numerous hydroxyl groups it can serve as a reducing agent. Gallic acid is also appreciated for its antioxidant properties, which are particularly well known in research on cytotoxicity against human cancer cells [34, 35].

In this paper, an aqueous extract of dog rose is used in the process of obtaining silver and gold nanoparticles. Using safe reagents brings the process in line with the principles of “green chemistry.” Using the proposed raw materials, such as wild dog rose, and specifically ellagic, gallic and ascorbic acids, and anthocyanins, does not entail the risk of a negative impact on the environment. There is a possibility of degradation of the unused product or raw materials, since aqueous solutions of the main reagents derived from renewable sources can be safely stored in the environment. Reaction conditions are also indifferent to environmental conditions. It is assumed that the process will be carried out at atmospheric pressure, which enables the efficient use of energy, and the process has mild effects on the environment.

## 2. Experimental

### 2.1. Materials

Silver nitrate, AgNO_3_, 99.90–99.99% pure, (Poch S.A. Company) and chloroauric acid, HAuCl_4_, 99.999% pure (Sigma Aldrich), were used as sources of the silver and gold ions. Sodium hydroxide (NaOH) (Poch S.A. Company) was used to adjust the pH. Water extract of the dried dog rose fruits was used as both the reducing and stabilising agent of the emerging nanosilver and nanogold suspensions. Disodium phosphate and potassium dihydrogen phosphate (V), in amounts of 0.5938 g and 0.4539 g, respectively, were used to prepare the phosphate buffer applied in electrochemical analysis. Subsequently, the salts were dissolved in water (50 mL) and mixed together at a ratio of 1 : 4 to give a buffer at pH 6.5 ± 0.05.

### 2.2. Methods

#### 2.2.1. Preparation of Aqueous Extracts of Dog Rose

Obtaining organic compounds from dried dog rose was performed by extraction in the aqueous phase using Soxhlet apparatus. In studies the dried fruits of dog rose were used. Fruits which served as sourcing material were mixed together and pulverized in a mortar. It was the step taken in order to unify the source material. In order to determine the conditions of the extraction process allowing an extract with the most favourable properties for the efficient reception of nanometals to be obtained, a number of extraction processes of different initial parameters were carried out. Using Statistica 10.0 a complete plan matrix was generated, based on which extraction was carried out. The ranges of input parameters are shown in Table 1.

Conducting the extraction process in Soxhlet apparatus set consists of weighing out a specified quantity of the raw material and placing it in a round bottom flask in the apparatus attachment. Water (300 g) is also placed in the flask as the solvent. The extraction process proceeds for a specified time. After extraction, the extraction flask contents are transferred to plastic containers and subsequently filtered. The supernatant was analysed spectrophotometrically (UV-VIS) in a wavelength range of *λ* = 300–1100 nm. Spectrophotometric analysis was performed in order to select those extracts in which characteristic absorption bands would not obscure the peaks originating from the nanosilver and nanogold. According to the literature data, this peak appears in a wavelength range from 400 to 500 nm for nanosilver and from 500 to 600 nm for nanogold. For the effective selection of spectra, statistical analysis techniques (agglomeration, k-means) were used. First, the number of clusters equal to a number of groups of objects (UV-Vis spectra) was defined, showing a maximum similarity within the clusters. At the same time, the objects belonging to different clusters are characterised by minimal mutual similarity. In order to accomplish this task, multivariate analysis using the Ward method was performed, assuming Euclidean distances between objects belonging to different clusters. As a result, a hierarchical tree showing the allocation of objects (spectra) for each cluster was created. The next step was to carry out spectral classification by the k-means method. This was intended to group the objects in a designated number of clusters and then average the value of each of them.

Using analytical techniques, the quantitative analysis of individual organic compounds was carried out. Total polyphenol content was determined in the supernatants by the Folin-Ciocalteu method [36]. The quantitative determination of ascorbic acid in the tested extracts was performed by the oximetric method [37]. The content of anthocyanins was determined by the Fuleki and Francis method [38]. The antioxidant activity of the obtained extracts was obtained by measuring the deactivation of free radicals (DPPH) and assessed using the Brand-Williams method [39].

The data was obtained in the form of percentages of extracted substances, and antioxidant activity was subjected to statistical analysis, by conducting tests on the significance of the influence of independent parameters (mass of plant material and extraction time) on the resulting values (percentage of extracted compounds and antioxidant activity) at a significance level of *α* = 0.05. The purpose of this was to determine the most favourable conditions for the extraction process so that the extracts obtained were characterised by the highest amount of extracted compounds. In keeping with the principles of “green chemistry,” the intention was to reduce the raw material and energy inputs. For this purpose, a response surface regression was defined. Generating this profile allows prediction of the dependent variable (percentage of extracted reducing and stabilising substances).

#### 2.2.2. Receiving of Nanometal Suspensions

The process of preparing the nanosilver and nanogold was carried out in the aqueous phase, and it was based on the chemical reduction method. Silver ions have been supplied by silver nitrate and gold ions by chloroauric acid. The concentrations of silver and gold salts were recalculated so that, in the course of research, the suspensions had a concentration of silver and gold of 50, 275, and 500 ppm. During the process, the independent parameters used were silver nitrate or chloroauric acid concentration, pH, and temperature of samples' incubation for the duration of the chemical reduction progress. The pH was adjusted with 0.1 M aqueous NaOH solution. Input data had three levels of volatility. In order to carry out the process in an effective way, the fractional plan 3^(3−1)^ was defined, which included 10 samples making up separate systems for each of the nanometals (one repetition in the centre of the plan). The realisation of the process obtaining nanomaterial involved mixing 76.8 mL of silver nitrate solution or chloroauric acid solution with 3.2 mL of extract on a magnetic stirrer. The values of given volumes were recalculated in order to obtain nanosilver and nanogold suspensions at concentrations of 50, 275, and 500 ppm. Under the conditions of continuous stirring, the pH was set to a determined value, and after one minute of further mixing the sample was transferred to a vessel at specified temperature conditions. A matrix of the fractional plan, which was the same for both nanometals, is included in Table 4.

Information on the process was obtained from UV-Vis analysis, after no further changes in the physicochemical properties of the samples were detected, which was identical with the lack of change in the appearance to the final absorption spectrum as compared with previous one spectrophotometric analysis was performed with a Rayleigh UV-1800 spectrophotometer. Spectrophotometric studies were performed at specified time intervals upon placing of the samples in the incubator. Spectrophotometric results in numerical form were subjected to statistical analysis techniques in order to identify the main clusters in which objects (UV-Vis spectra) were derived. The purpose of this step was to find the identical spectra to the spectra characteristic for nanometric silver and gold.

Similarly, as with the analyses of the spectra of the extracts, the purpose of this operation was to group the spectra obtained so that spectra inside the cluster showed the greatest similarity, while the greatest variety of the objects characterised different clusters. Multivariate analysis was performed using the Ward method, assuming Euclidean distances between objects belonging to different clusters. During the course of analysis, a hierarchical tree of allocated objects (spectra) for each cluster was generated.

The resulting suspensions were analysed to define particle size and electrokinetic potential values. Analysis of the size of silver and gold nanoparticles and the stability of the suspension was conducted using dynamic light scattering (DLS) on a Zetasizer Nano Malvern. As a result of the tests, a histogram that graphically illustrates the percentage of nanoparticles of a given size was obtained. Furthermore, values of electrokinetic potential *ζ*, which is an expression of the stability of the suspension, were obtained. The data were analysed statistically. It was found that the input variables (AgNO_3_ or HAuCl_4_ concentration, pH, temperature) have the largest statistically significant impact of the output variables (size and zeta potential of nanoparticles) at *α* = 0.05.

#### 2.2.3. Receiving of the Solid Form of Nanosilver

In order to obtain a solid form of nanosilver and nanogold, the obtained suspensions were centrifuged using a Sorvall Ultracentrifuge Thermo Scientific & WX Ultra Micro-Ultra Series. The parameters used were 50 000 r/min., 15 min. run time, and 20°C temperature. After centrifugation the supernatant was discarded and the separated solid was dried under atmospheric conditions. The resulting powder samples were subjected to microscopic analysis using scanning electron microscopy (SEM) (LEO Electron Microscopy Ltd.).

## 3. Results and Discussion

### 3.1. Extract with Most Favourable Properties

**Figure 1:**
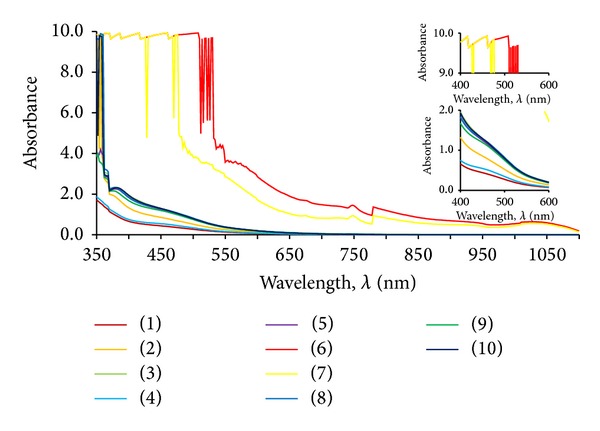
Set of UV-Vis spectra of dog rose extracts.

As a result of spectrophotometric analysis, UV-Vis spectra were obtained which characterised the dog rose extracts. Spectrophotometric spectra of the series extracts are shown in Figure 1.

**Figure 2:**
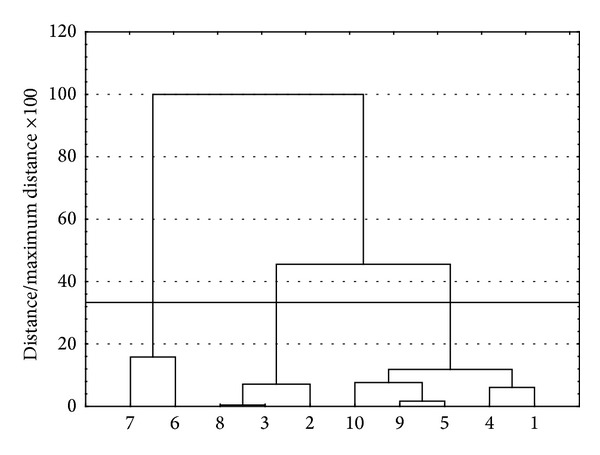
Hierarchical tree showing the assignment of objects (spectra) for each cluster (Ward method, Euclidean distances).

In order to classify the obtained spectra data, they were analysed by multivariate analysis. It was the step taken in order to reveal the group of these specific spectra which would not overlap with the spectra originating from nanosilver or nanogold suspensions. This analysis helped to clarify the whole spectra data and to choose the specific extract whose UV-Vis curve does not occur in the region which is characteristic for nanometals particles. Data obtained throughout the whole range of wavelength shown in Figure 1 were used in the statistical analysis. The results are shown in Figure 2.

Assuming strict Sneath (33%) criterion, objects can be divided into three focus spectra. This means that the resulting spectrum can be divided into three major groups, with a high spectra similarity within the group and a considerable difference between objects belonging to different groups.

**Figure 3:**
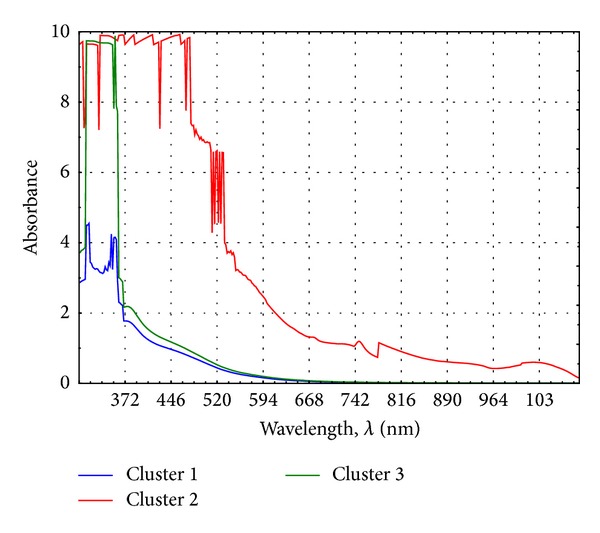
Chart of mean clusters containing spectra of dog rose extracts.

The spectra were also classified by the k-means method. This was intended to group objects in a designated number of clusters and average the value of each of them, as shown graphically in Figure 3.

During the analysis it was found that the UV-Vis spectrum of the extract belonging to the third cluster is characterised by the most favourable optical properties. The averaged spectrum belonging to the third cluster does not occur in the area in which the characteristic peaks for nanosilver and nanogold appear. In comparison with the spectra of extracts belonging to clusters 1 and 2, the absorbance values are low and do not constitute an obstacle to the optical spectra of the nanometals.

Observation shows that with an increase in both the process time and the dry weight of the plant material, the spectra of obtained extracts are characterised as least significant in terms of utility in the preparation of the nanometal clusters. Increasing the weight of the plant material and extraction time leads to higher extract concentration and higher absorbance values in the spectra characterising them. Considering the optical aspects, the concentrated extracts should be diluted or the extraction process should be led with the least amount of dry dog rose plant and in the shortest possible time.

For a measurement to determine the composition of the selected plant material, the following compounds were considered: polyphenols, ascorbic acid, and anthocyanins. By measuring the effectiveness of the inactivation of free radicals, the power of antioxidants contained in each extract was also examined. The results of the analysis, as well as the percentage of extractable substances, are presented in Table 2.

The analysis of these results is presented in Table 3, in the form of tests of significance on the impact of independent parameters (weight of plant material and extraction time) on the resulting values, which is the percentage of extracted substances and antioxidant activity at a level of significance *α* = 0.05.

On the basis of assessing the significance of the effect of two independent variables on the percentage of extracted substances, the following were found to be significant: the mass of plant material, for polyphenols and ascorbic acid and the mass of plant material and time of extraction, for anthocyanins. These variables showed a significance of *α* = 0.05. For the determination of antioxidant properties, no input variable was statistically significant.

**Figure 4:**
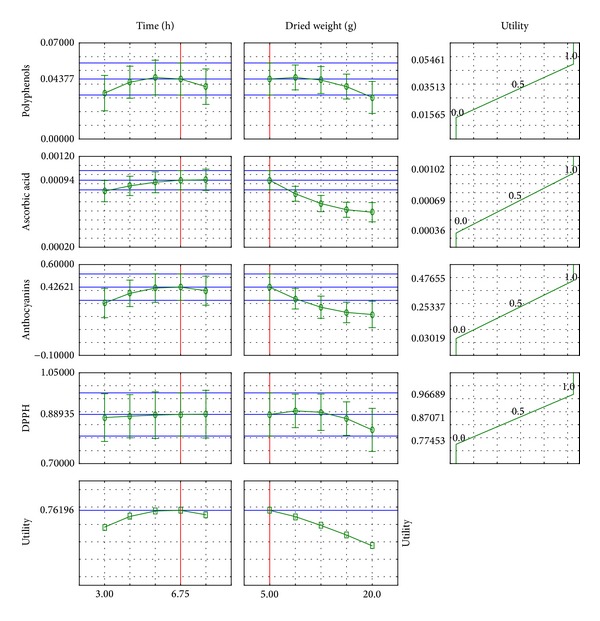
Response surface regression profile for dog rose extract.

Response surface regression was also defined, from which it is possible to predict the value of the output variables, assuming a value of the input data. A profile is shown in Figure 4.

For this profile, a relationship between the amount of extracted substances and the parameters of extraction can be determined. With an increase in extraction time the percentage of extracted compounds increases, which results in a more concentrated extract being obtained. Antioxidative activity also increases due to a larger number of compounds with antioxidant activity. The profile suggests that an increase in the weight of the plant material does not lead to a larger number of tested compounds. This may be due to the fact that only a prolonged extraction time and increasing the duration of the process improves the efficiency, in other words from a small amount of dog rose enough compounds may be extracted.

The values of input variables for which the output variables are understood to be the most effective are as follows: mass = 5.0 [g] and time = 6.75 [h].

After determining the conditions of the extraction process, extraction was performed using dog rose with the chosen input values. A comparison of the values of the percentage of extracted substances against the antioxidant activity of the extracts labelled after the extraction process is presented in Table 4.

Statistical analysis showed that the spectrophotometric spectrum of the selected extract belongs to the third cluster, so that it does not constitute an optical obstacle to the spectra characteristic of nanosilver and nanogold.

### 3.2. Nanometal Suspensions

#### 3.2.1. Nanosilver

**Figure 5:**
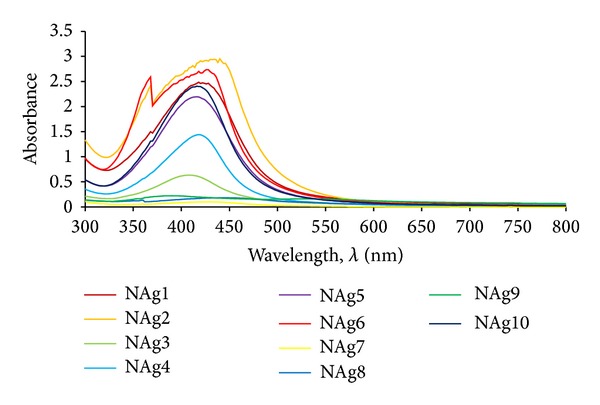
Spectrophotometric spectra obtained for a suspension of silver based on an extract of dog rose.

*UV-Vis Spectrophotometry*. All received suspensions were analysed spectrophotometrically, and 10 UV-Vis spectra were obtained. These are summarised in Figure 5.

**Figure 6:**
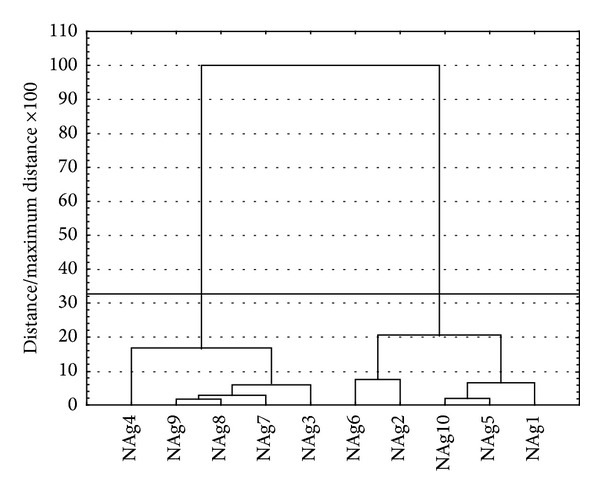
Hierarchical tree showing the assignment of objects (spectra of nanosilver) for each cluster.

The UV-Vis spectra data obtained for silver nanosuspensions were classified by the statistical analyses. It helped to see the differences in peaks maximum between samples obtained in different conditions. Spectrophotometric data were subjected to multivariate analysis using the Ward method and assuming Euclidean distances. Data obtained throughout the whole range of wavelength shown in Figure 5 were used in the statistical analysis. As a result, a hierarchical tree was generated, which is shown in Figure 6.

**Figure 7:**
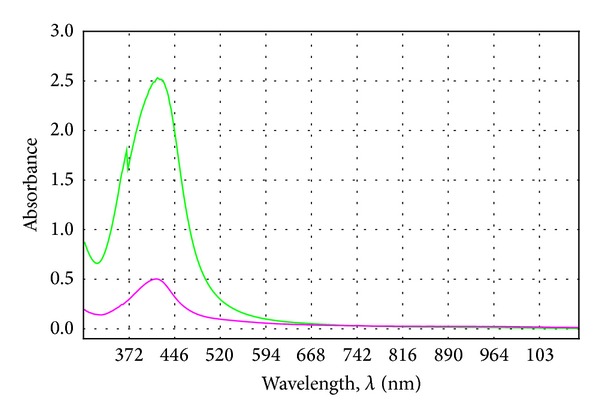
Chart of average clusters containing NAg suspensions spectrum.

Taking a more stringent criterion (33%), the objects can be divided into two clusters of spectra. This means that the spectrum within the cluster has a high similarity, and the objects belonging to different clusters have significant differences. With the k-means method, objects (spectra) are grouped into clusters and the average amount of each cluster is determined, as shown in Figure 7.

In the UV-Vis spectra obtained for systems 1, 2, 5, 6, and 10 (cluster 2) an intensive peak originating from the nanosilver can be observed, whose maximum is set in the range from 400 to 450 nm. A chart of average values (Figure 7) suggests that the spectrum belonging to the first cluster characterises the suspensions in which nanosilver could not successfully be obtained. In Table 5, spectra belonging to each cluster is marked with colour. Nanosilver suspensions were fully formed in systems in which a lower concentration of silver nitrate was used, so that the ratio of the amount of stabilising and reducing compounds to silver ions was sufficient, and reduction occurred successfully. In the case of the lowest concentration of silver ions it is not necessary to increase the pH in order to effectively carry out the process.

For a higher concentration of silver salt, the pH of the reaction mixture should be increased, because the alkaline environment enhances the action of reducers, and consequently a lower amount of them is sufficient to reduce a larger amount of silver ions. Measuring the size of nanoparticles and nanosilver suspensions' stability.

The results of the analyses of nanoparticles size (*d*, nm) and the stability of the suspension (*ζ*, mV) are presented in Table 6.

It should be noted that the suspensions belonging to cluster 2 are characterised by a higher degree of conversion, in turn characterised by the known absorbance value and the spectra of the other samples being less intense, which translates into a lower degree of conversion. In Table 5 a time value (*t* [h]) after which no further changes were observed in the appearance of the spectrophotometric spectra of the products obtained is shown.

**Figure 8:**
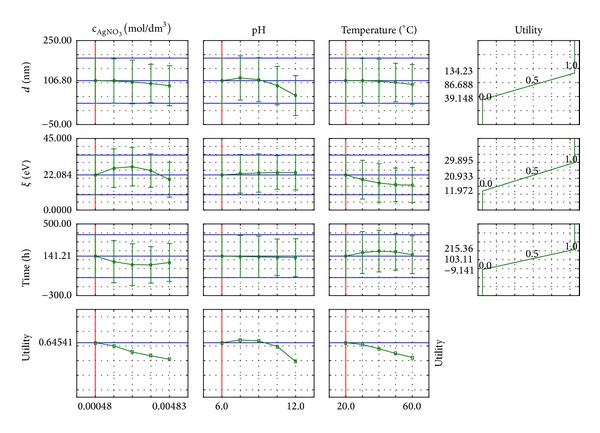
Profile of response surface regression predicted for nanosilver suspensions obtained based on dog rose extract.

The results were statistically analysed in order to carry out response surface regression. Profiles were determined for the lowest values of the input quantities, such as a silver salt concentration, pH, and temperature. Profiles of predicted dependent variables (*d*, *ζ*, and *t*) are shown in Figure 8.

Regarding the profiles, the course of the size distribution of the silver nanoparticles and the stability of the suspension, the dependence on the size of the input may be predicted.

For the assessment a wide range of silver nitrate concentrations were used. The highest concentration was 10 times higher than the lowest one, but the suspensions of nanometric silver were only able to receive the two lowest concentrations of silver ions sources (Figure 8). In these cases, the amount of reducer and stabiliser contained in the extract was sufficient for an efficient process execution. With the increase in silver nitrate concentration, the particles' size is slightly reduced. This is due to the predominance of the degree of intensification in chemical reduction compared to the reduced effectiveness of the stability in terms of increased concentration of AgNO_3_. Then, a larger number of smaller nanoparticles with a higher degree of stability appear.

Increasing the pH favours the receiving nanoparticles, which are characterised by being smaller in size, because with the increase in the amount of hydroxyl ions (increase in pH value) the reduction reaction is favoured and the partial processes overlap with a higher efficiency. The presence of OH^−^ ions affects the activation of the reducing compounds, and with an increasing pH their activity increases, and a greater number of smaller embryos is created. This state is in direct relation to the increasing value in electrokinetic potential—smaller particles are more stable.

With an increasing temperature, the chemical reduction process takes place more intensively, because the mobility of the ions is increased and the reducing agent is more highly active. As a result there is greater amount of smaller particles created per time unit, and with a suitably selected amount of stabilising material the suspension obtained is stable over time. The increase in temperature can also cause more intensive movement of particles that collide together in larger agglomerates and fall.

The fact that the extract includes a lower amount of reducer and stabiliser results in the stability of the particles being lower, as shown by the decreasing value of electrokinetic potential.

Increasing the silver nitrate concentration reduces the time needed to carry out the process. The supplied amount of reducing and stabilising agents is sufficient to result in a shorter time for effective implementation of the chemical reduction process and the stabilisation of the resulting suspension. This involves the preparation of smaller nanosilver particles.

**Figure 9:**
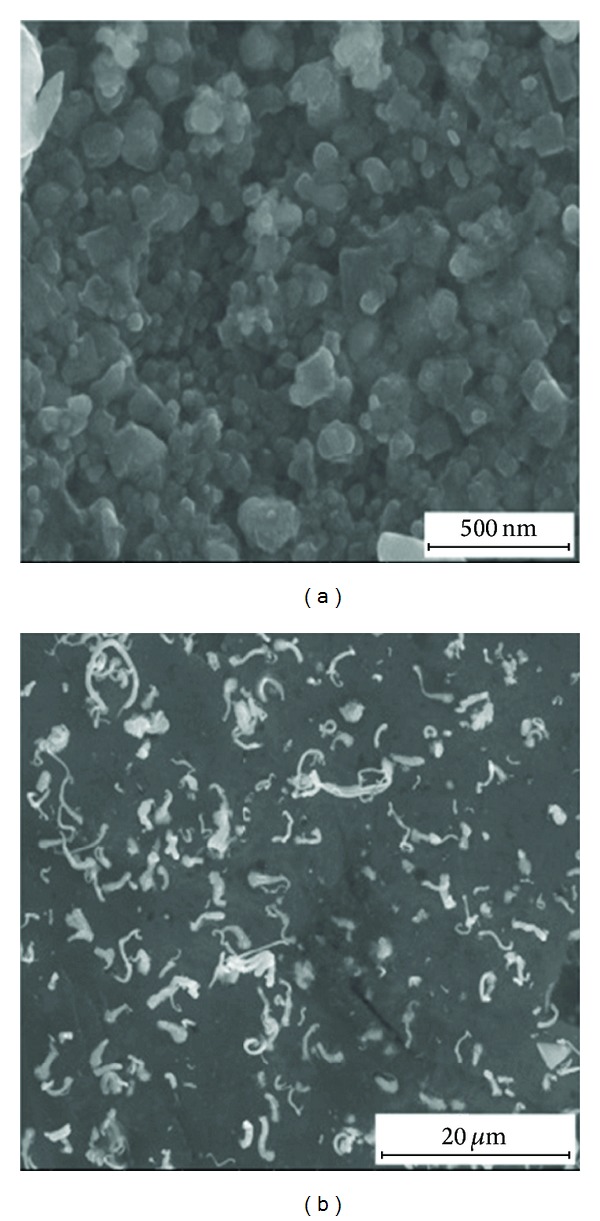
SEM photographs of nanosilver prepared on the basis of the dog rose extract: (a) system 1, (b) system 7.

*Scanning Electron Microscopy*. Scanning electron microscopy revealed the shapes of the silver nanoparticles in the obtained samples. Photographs of selected samples are shown in Figure 9.

The resulting pictures show that the silver nanoparticles are characterised by a three-dimensional shape. In the first sample, the nanoparticles are present with an irregular shape, while sample 7 consists of shaving-like particles, which form an irregular structure.

#### 3.2.2. Nanogold

**Figure 10:**
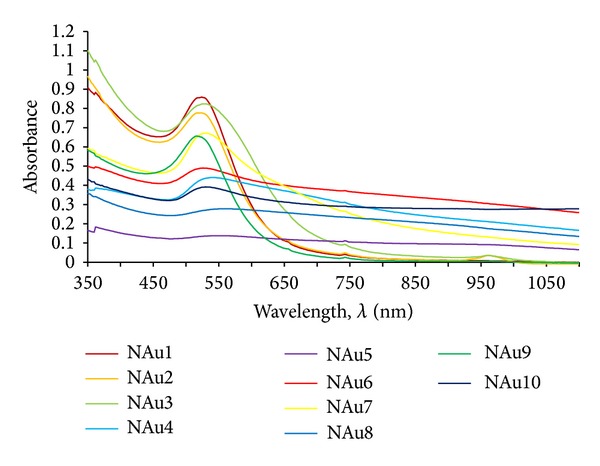
Spectrophotometric spectra of nanogold suspensions obtained based on dog rose extract.

*UV-Vis Spectrophotometry*. A series of spectra of obtained nanogold suspensions was obtained, the set of which is shown in Figure 10.

**Figure 11:**
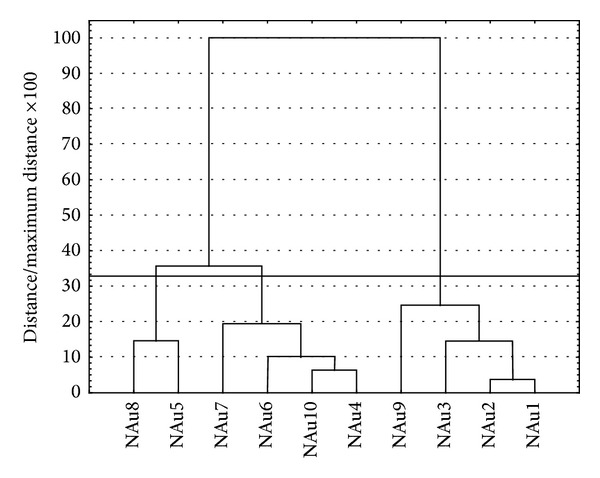
Hierarchical tree showing the assignment of objects (spectra of nanogold) for each cluster.

In order to interpret the results they were subjected to statistical analysis. The UV-Vis spectra data obtained for gold nanosuspensions were classified by the statistical analyses. It helped to see the differences in peaks maximum between samples obtained in different conditions. Data obtained throughout the whole range of wavelength shown in Figure 10 were used in the statistical analysis. A hierarchical tree of the multivariate analysis using the Ward method, assuming Euclidean distances, is shown in Figure 11.

**Figure 12:**
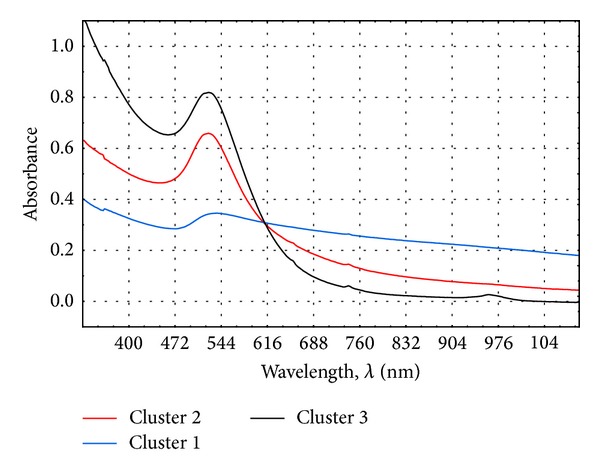
Chart of average clusters containing NAu suspension spectrum.

Assuming strict Sneath criterion (33%), the collection of objects can be divided into three clusters of spectra. This means that the resulting spectra can be divided into three major groups, with a high similarity of spectra within the groups and a considerable difference between the objects belonging to different groups. Using the method of k-means, the spectra are assigned to clusters and the average value of each is determined (Figure 12).

Basing on the above results it was found that the UV-Vis spectra belonging to clusters 2 and 3 are derived from a suspension containing gold nanoparticles (systems 1, 2, 3, 7, and 9). Table 4 lists the spectra belonging to each cluster, marking them with colour. These suspensions were prepared under conditions of lowest concentration of gold salt (systems 1, 2, and 3), and sample 9 was obtained at the highest pH value. When using higher concentrations of chloroauric acid, the samples were characterised by a lower and broader spectrum peak, which indicates the formation of larger particles with polymodal size distribution. The lowest concentration of the gold salt allowed the effective reduction of ions and stabilisation of the resulting suspension. After increasing the concentration of the source of gold ions, in order to obtain gold nanoparticles, it is necessary to increase the pH, as an alkaline environment favours the action of chemical reduction.

*Measuring the Size of Nanoparticles and Stability of the Nanogold Suspension.* The results of nanoparticles size measurement (*d*, nm) and the stability of the suspension (*ζ*, mV) are provided in Table 7.

**Figure 13:**
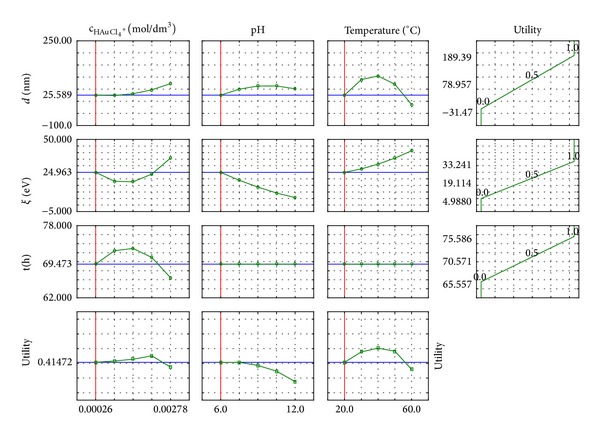
Response surface regression of output variables for nanogold suspensions obtained based on dog rose extract.

Table 7 also lists the value of time (*t* [h]) after which no further changes in the appearance of the spectra of obtained products were noticed. As in the case of the suspension of nanosilver, the obtained data were analysed statistically in order to determine the response surface regression profiles. Profiles were determined for the minimum values of the input quantities, such as gold salt concentration, pH, and temperature. The response surface regression of the output variables (*d*, *ζ*, and *t*) are shown in Figure 13.

It is important to identify those chloroauric acid concentrations at which it is still possible to obtain a stable suspension of nanogold. In these studies, stable products could be obtained with a maximum concentration of HAuCl_4_ at 0.001454 mol/dm^3^. Increasing the concentration of chloroauric acid leads to the suspension containing more gold nanoparticles. This relationship is also reflected by the shift in the maximum peak derived from the resultant suspension towards the infrared (Figure 12). These results are important for practical reasons, as in the case of gold nanoparticles production on an industrial scale, lower concentration values are used. Indirectly, this is related to the effectiveness of the stabilisation process. Increasing the concentration of gold ions or reducing the molar ratio of the stabilising agents for gold ions causes the stabiliser efficiency to decrease, which leads to an agglomeration of embryos and the creation of larger particles.

The resulting profiles suggest that increasing the pH causes an increase in the size of gold nanoparticles and the reduction in suspension stability. Here, attention should be paid to the mechanism of the reduction process occurring in the reaction within the mixtures of different pH values. Referring to the literature data [40] the structure of the complex ions derived from the chloroauric acid depends on the number of hydroxyl ions present in the mixture. Its form depends on the pH value, which is shown in Table 7.

With the increase in the intensity of the Cl^−^ ion exchange of an OH^−^ group, the stability of the complex increases. Thus it is more difficult for the reducing agent to reduce metallic gold. Therefore, in conditions of higher pH the process of chemical reduction is weaker. Thus there is a lower number of embryos that agglomerate and the resulting suspension contains large gold nanoparticles. The decreasing value of the electrokinetic potential value is related to their proliferation. When the size of nanoparticles exceeds the limiting value, the Brownian motion is weaker and the agglomerated particles fall.

The effect of temperature on the size of the nanoparticles is nonlinear. This is due to the slow formation of embryos which at lower temperature are not stabilised effectively, and by connection with each other they increase in size. Under conditions of higher temperature, an intensification in the reduction process occurs and a greater number of embryos are characterised by a smaller size.

Taking into consideration the sample incubation time, after which no further changes in the appearance of the spectra were observed thus marking the completion of the process, it was concluded that in the case of the lowest chloroauric acid concentrations the shortest incubation time of samples that allows implementation of the process is sufficient. This is due to the favourable ratio of the reducing and stabilising agents to the gold ions. As the concentration of gold ion source increases, changes in the appearance of the spectra slowly cease appearing, thus indicating the progressive agglomeration of the resulting nanoparticles, due to ineffective stabilisation since the ratio of the stabiliser to gold ions is lower.

**Figure 14:**
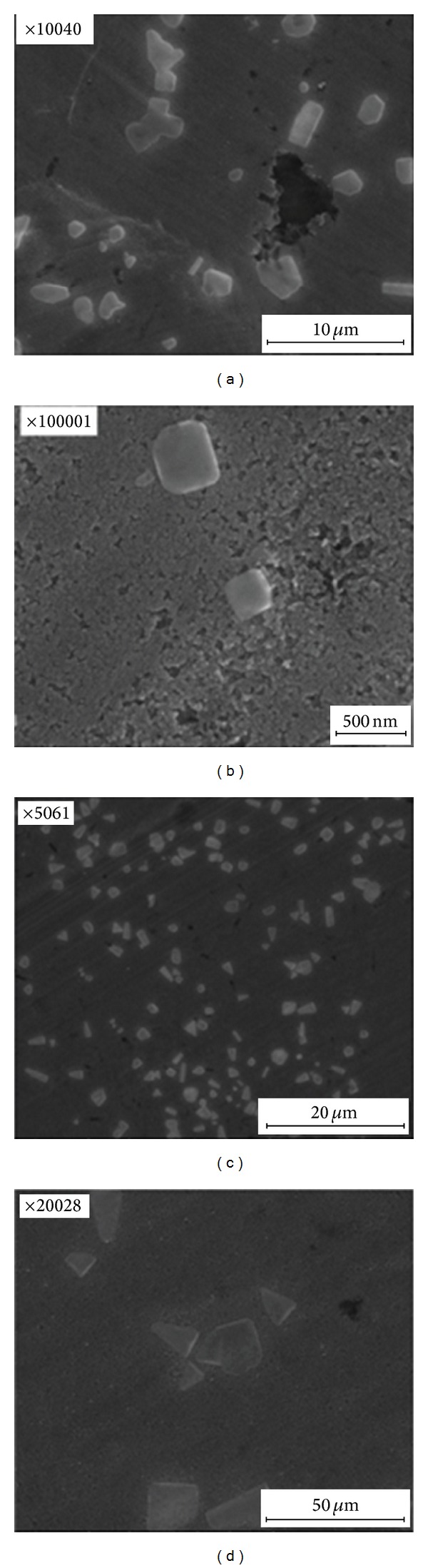
SEM images of nanogold obtained on the basis of dog rose extract: (a) and (b), system 6, (c), (d), system 7.

*Scanning Electron Microscopy. *Microphotographs obtained were used to assess the shape of the nanoparticles of gold contained in the samples. Pictures of the two samples of powder are shown in Figure 14.

Images of the obtained product on the basis of dog rose extract in system 6 show that the shape of the nanoparticles is rectangular, and in the case of the system 7 it is tetrahedral. Shape variability can be explained by the variety of process conditions when receiving the nanogold suspensions. The suspension containing particles of rectangular shape has been obtained under the lowest temperature conditions, and the three-dimensional particles were obtained by the process carried out in medium values of temperature and gold ion concentration, and with the lowest pH value.

## 4. Conclusions

As a result of this work, based on a quantitative analysis of polyphenolic compounds, ascorbic acid, and anthocyanins as well as the ability of inhibition of free radicals, the most favourable composition of an extract and parameters of extraction of a natural plant material, dried dog rose, were determined. A method for preparing nanosilver and nanogold with an aqueous extract of dog rose as the natural source of the reducing and stabilising agents was developed. The extract was stable for at least two weeks. During this time, no visual changes in appearance of extracts were observed. If any changes arise, then mold growth could be expected. It would have precluded the use of the extract as a raw material in the preparation of nanosilver or nanogold particles. The influence of the process parameters (the concentration of the source of metal ions, pH and temperature) on the properties of the obtained suspension was determined. The method can be successfully used as an ecological process for the preparation of nanosilver and nanogold. The dependence of reduction process in obtaining nanometals particles on the environmental conditions in which the plant was growing up is a very important issue. Other studies should be held whose aim would be to reveal the influence of the plant age, the area of it growth and, other factors on the compositional profile. Such studies would be helpful in standardization of the whole process.

## Figures and Tables

**Table 1:** Plan matrix for extraction processes.

No. of system	Input variables	
	Time (h)	Mass of material source (g)
1	3.00	5.00
2	3.00	12.50
3	3.00	20.00
4	5.50	5.00
5	5.50	12.50
6	5.50	20.00
7	8.00	5.00
8	8.00	12.50
9	8.00	20.00
10	5.50	12.50

**Table 2:** Results of the quantitative analysis and effectiveness of antioxidant in dog rose extracts.

No. of system	Polyphenols		Ascorbic acid		Anthocyanins		DPPH (%)
	mg/mL	Percentage of extracted polyphenols (%)	mg/mL	Percentage of extracted ascorbic acid (%)	mg/1 g dried weight	Percentage of extracted anthocyanins (%)	
1	0.4546	0.02727	14.08	0.000844	3.7934	0.2276	92.46
2	1.3357	0.03258	21.12	0.000506	6.9677	0.1672	87.76
3	1.7665	0.02649	32.85	0.000492	9.9871	0.1498	77.92
4	0.7597	0.04558	14.08	0.000844	8.2065	0.4924	83.17
5	1.6640	0.03993	29.33	0.000703	10.297	0.2471	92.13
6	1.7956	0.02693	38.89	0.000598	10.916	0.1637	82.95
7	0.7274	0.04364	16.43	0.000985	6.6968	0.4018	89.84
8	1.4072	0.03377	29.33	0.000703	10.606	0.2546	85.46
9	1.5302	0.02295	35.20	0.000528	11.729	0.1759	86.78
10	2.1723	0.05213	26.99	0.000647	10.568	0.2536	92.24

**Table 3:** Tests of significance for the extracted substances (ANOVA).

	Polyphenols			Ascorbic acid			Anthocyanins			DPPH		
	Degrees of freedom	*F*	*P*	Degrees of freedom	*F*	*P*	Degrees of freedom	*F*	*P*	Degrees of freedom	*F*	*P*
Absolute term	**1**	**281.5305**	**0.000014**	**1**	**1035.819**	**0.000001**	**1**	**999.4516**	**0.000001**	**1**	**3138.275**	**0.000000**
Mass	**2**	**18.8011**	**0.004719**	**2**	**54.242**	**0.000407**	**2**	**22.4283**	**0.003185**	2	0.060	0.942846
Time	2	2.2616	0.199739	2	3.521	0.111089	**2**	**11.3832**	**0.013760**	2	1.873	0.247096
Error	5			5			5			5		

**Table 4:** Matrix of the fractional plan for obtaining nanosilver and nanogold.

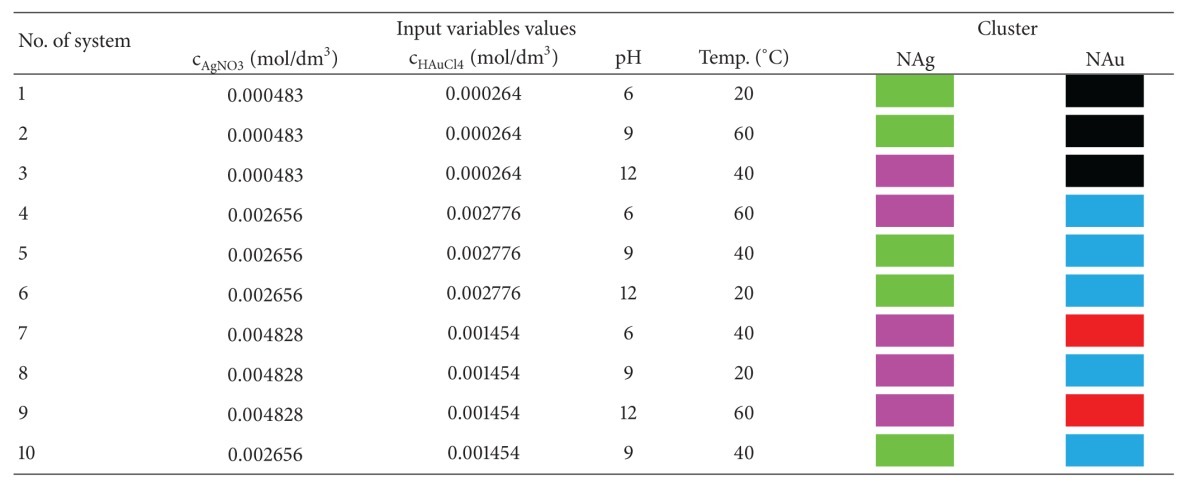

**Table 5:** Results of the average particle size and stability of nanosilver suspension.

No. of system	*d* (nm)	*ξ* (mV)	*t* (h)
1	106.8	−22.1	141.0
2	101.0	−18.3	163.0
3	47.44	−17.2	163.0
4	84.92	−19.5	44.50
5	121.3	−21.7	44.50
6	52.79	−30.1	44.50
7	90.77	−15.4	141.5
8	88.93	−19.0	45.00
10	86.27	−25.1	141.0

**Table 6:** Results of the average particle size and stability of the nanogold suspension.

No. of system	*d* (nm)	*ξ* (mV)	*t* (h)
1	25.55	−24.9	69.5
2	24.06	−30.0	69.5
3	130.9	−11.8	69.5
6	98.85	−16.4	66.5
7	109.3	−23.7	73.0
9	17.24	−14.6	73.0
10	146.8	−12.4	73.0

**Table 7:** Specification of Au (III) complexes formed after the addition of NaOH to a solution of chloroauric acid.

pH ranges	Complex
0.0–3.0	AuCl_4_^−^·HCl
3.1–7.0	AuCl_4_^−^
7.1–9.0	AuCl_3_OH^−^
9.1–10.0	AuCl_2_(OH)_2_^−^
10.1–11.0	AuCl(OH)_3_^−^
11.1–13.5	Au(OH)_4_^−^

## References

[1] Brown CL, Bushell G, Whitehouse MW (2007). Nanogold-pharmaceutics: (i) the use of colloidal gold to treat experimentally-induced arthritis in rat models; (ii) characterization of the gold in Swarna bhasma, a microparticulate used in traditional Indian medicine. *Gold Bulletin*.

[2] Horisberger M, Rosset J (1977). Colloidal gold, a useful marker for transmission and scanning electron microscopy. *Journal of Histochemistry and Cytochemistry*.

[3] Alanazi FK, Radwan AA, Alsarra IA (2010). Biopharmaceutical applications of nanogold. *Saudi Pharmaceutical Journal*.

[4] Pulit J, Banach M, Kowalski Z (2013). Chemical reduction as the main method for obtaining nanosilver. *Journal of Computational and Theoretical Nanoscience*.

[5] Otari SV, Patil RM, Nadaf NH, Ghosh SJ, Pawar SH (2012). Green biosynthesis of silver nanoparticles from an actinobacteria Rhodococcus sp. *Materials Letters*.

[6] Iravani S (2011). Green synthesis of metal nanoparticles using plants. *Green Chemistry*.

[7] Krpetić Ž, Scarì G, Caneva E, Speranza G, Porta F (2009). Gold nanoparticles prepared using cape aloe active components. *Langmuir*.

[8] Shankar SS, Rai A, Ahmad A, Sastry M (2011). Biosynthesis of silver nanoparticles using Calotropis gigantean leaf. *African Journal of Basic & Applied Sciences*.

[9] Mondal AK, Mondal S, Samanta S, Mallick S (2011). Synthesis of ecofriendly silver nanoparticle from plant latex used as an important taxonomic tool for phylogenetic interrelationship. *Advances in Bioresearch*.

[10] Li S, Shen Y, Xie A (2007). Green synthesis of silver nanoparticles using Capsicum annuum L. extract. *Green Chemistry*.

[11] Gnanadesigan M, Anand M, Ravikumar S (2011). Biosynthesis of silver nanoparticles by using mangrove plant extract and their potential mosquito larvicidal property. *Asian Pacific Journal of Tropical Medicine*.

[12] Dwivedi AD, Gopal K (2010). Biosynthesis of silver and gold nanoparticles using Chenopodium album leaf extract. *Colloids and Surfaces A*.

[13] Bar H, Bhui DK, Sahoo GP, Sarkar P, Pyne S, Misra A (2009). Green synthesis of silver nanoparticles using seed extract of Jatropha curcas. *Colloids and Surfaces A*.

[14] Kasthuri J, Veerapandian S, Rajendiran N (2009). Biological synthesis of silver and gold nanoparticles using apiin as reducing agent. *Colloids and Surfaces B*.

[15] Begum NA, Mondal S, Basu S, Laskar RA, Mandal D (2009). Biogenic synthesis of Au and Ag nanoparticles using aqueous solutions of Black Tea leaf extracts. *Colloids and Surfaces B*.

[16] Martinez-Castanon GA, Niño-Martínez N, Martínez-Gutierrez F, Martínez-Mendoza JR, Ruiz F (2008). Synthesis and antibacterial activity of silver nanoparticles with different sizes. *Journal of Nanoparticle Research*.

[17] Owen RW, Giacosa A, Hull WE, Haubner R, Spiegelhalder B, Bartsch H (2000). The antioxidant/anticancer potential of phenolic compounds isolated from olive oil. *European Journal of Cancer*.

[18] Lotito SB, Frei B (2004). Relevance of apple polyphenols as antioxidants in human plasma: contrasting in vitro and in vivo effects. *Free Radical Biology and Medicine*.

[19] Scalbert A, Johnson IT, Saltmarsh M (2005). Polyphenols: antioxidants and beyond. *The American Journal of Clinical Nutrition*.

[20] Bozan B, Sagdullaev BT, Kozar M, Aripov KN, Baser KHC (1998). Comparison of ascorbic and citric acid contents in Rosa Canina L. fruit growing in the Central Asian region. *Chemistry of Natural Compounds*.

[21] Kazaz S, Baydar H, Erbas S (2009). Variations in chemical compositions of Rosa damascena Mill, and Rosa canina L. Fruits. *Czech Journal of Food Sciences*.

[22] Kilicgun H, Dehen A (2009). In vitro antioxidant effect of Rosa canina in different antioxidant test systems. *Pharmacognosy Research*.

[23] Ghazghazi H, Miguel MG, Hasnaoui B (2010). Phenols, essential oils and carotenoids of Rosa canina from Tunisia and their antioxidant activities. *African Journal of Biotechnology*.

[24] Kilicgun H, Altiner D (2010). Correlation between antioxidant effect mechanisms and polyphenol content of Rosa canina. *Pharmacognosy Magazine*.

[25] Niki E (1991). Action of ascorbic acid as a scavenger of active and stable oxygen radicals. *American Journal of Clinical Nutrition*.

[26] Padayatty SJ, Katz A, Wang Y (2003). Vitamin C as an antioxidant: evaluation of its role in disease prevention. *Journal of the American College of Nutrition*.

[27] Kazaz S, Baydar H, Erbas S (2009). Variations in chemical compositions of Rosa damascena Mill, and Rosa canina L. Fruits. *Czech Journal of Food Sciences*.

[28] Roman I, Stănilă A, Stănilă S (2013). Bioactive compounds and antioxidant activity of Rosa canina L. biotypes from spontaneous flora of Transylvania. *Chemistry Central Journal*.

[29] Nojavana S, Khaliliana F, Kiaiec FM, Rahimic A, Arabanianc A, Chalavia S (2008). Extraction and quantitative determination of ascorbic acid during different maturity stages of Rosa canina L. fruit. *Journal of Food Composition and Analysis*.

[30] Nowak R, Gawlik-Dziki U (2007). Polyphenols of Rosa L. leaves extracts and their radical scavenging activity. *Zeitschrift fur Naturforschung C*.

[31] Hvattum E (2002). Determination of phenolic compounds in rose hip (Rosa canina) using liquid chromatography coupled to electrospray ionisation tandem mass spectrometry and diode-array detection. *Rapid Communications in Mass Spectrometry*.

[32] Meyer AS, Heinonen M, Frankel EN (1998). Antioxidant interactions of catechin, cyanidin, caffeic acid, quercetin, and ellagic acid on human LDL oxidation. *Food Chemistry*.

[33] Falsaperla M, Morgia G, Tartarone A, Ardito R, Romano G (2005). Support ellagic acid therapy in patients with hormone refractory prostate cancer (HRPC) on standard chemotherapy using vinorelbine and estramustine phosphate. *European Urology*.

[34] Fiuza SM, Gomes C, Teixeira LJ (2004). Phenolic acid derivatives with potential anticancer properties—a structure-activity relationship study. Part 1: methyl, propyl and octyl esters of caffeic and gallic acids. *Bioorganic and Medicinal Chemistry*.

[35] Stoner GD, Mukhtar H (1995). Polyphenols as cancer chemopreventive agents. *Journal of Cellular Biochemistry*.

[36] Strzelecka H, Kaminska J, Kowalski J, Walewska E (1982). *Chemiczne Metody Badań Roślinnych Surowcow Leczniczych—Podręcznik Dla Studentow Farmacji*.

[37] Hughes DE (1983). Titrimetric determination of ascorbic acid with 2,6-dichlorophenol indophenol in commercial liquid diets. *Journal of Pharmaceutical Sciences*.

[38] Fuleki T, Francis FJ (1968). Quantitative methods for anthocyanins. Extraction and determination of total anthocyanin in cranberries. *Journal of Food Science*.

[39] Bondet V, Brand-Williams W, Berset C (1997). Kinetics and mechanisms of antioxidant activity using the DPPH• free radical method. *LWT—Food Science and Technology*.

[40] Morrow BJ, Matijević E, Goia DV (2009). Preparation and stabilization of monodisperse colloidal gold by reduction with aminodextran. *Journal of Colloid and Interface Science*.

